# Meducide - Towards a new conceptual framework for the destruction of medical education in armed conflicts

**DOI:** 10.12688/mep.21604.1

**Published:** 2026-06-18

**Authors:** Suhad Daher-Nashif

**Affiliations:** 1 Keele University School of Medicine, Keele, England, UK

**Keywords:** Medical education, War and armed conflict, Health workforce, Public health, Teaching hospitals, Accreditation, Humanitarian health

## Abstract

Wars and armed conflicts are widely recognised as a threat to health systems, yet the destruction of medical education is still too often treated as a secondary educational disruption. This opinion article argues that attacks on medical schools, teaching hospitals, clinical placements, accreditation systems and faculty pipelines should be understood as a distinct form of long-term public health harm. I propose the term "meducide" to describe the systematic destruction, dismantling or incapacitation of medical education during war, whether by direct attack or by structural conditions that render medical training impossible. The concept builds on existing scholarship on attacks on education, structural violence and health workforce fragility, while highlighting what is distinctive about medical education. It captures not only the destruction of medical schools and teaching hospitals but also the broader erosion of the conditions necessary to train future healthcare. Therefore, it sits at the intersection of higher education, health service delivery, professional regulation and the future health workforce. Naming Meducide does not replace careful empirical documentation or legal assessment of intent. Rather, it offers a precise conceptual language for recognising a recurring pattern: when medical education is destroyed, the effects extend beyond students and universities to patient care, population health, post-conflict recovery and health system sovereignty. The article calls for medical education to be documented separately in conflict monitoring, protected as critical civilian and health infrastructure, included in humanitarian and reconstruction planning, and supported through global recognition, cross borders faculty networks, conflict-adapted curricula and sustainable clinical training pathways.

## Introduction

Wars damage health systems through visible and immediate violence; hospitals are bombed or occupied, health workers are killed or displaced, medicines and equipment disappear, and patients lose access to care. ^1^ It also damages health systems through a slower mechanism that remains less visible in medical education scholarship, including medical schools, teaching hospitals, clinical placements, laboratories, libraries, licensing examinations and faculty communities are not peripheral academic assets. ^2, 3^ These are major infrastructure for reproducing safe clinical practice.

Medical education is unusually dependent on stable institutions. I.e., a medical degree isn’t limited to lectures, examinations or online modules. It requires supervised clinical exposure, regulated progression, assessment of competence, mentorship, and teaching hospitals. Therefore, when these structures collapse, the result is not only interrupted education, but weakening of future clinical capacity, that directly damages populations health and healing during and after conflicts.

The existing literature already shows how conflict massively disrupts medical education in Iraq, Syria, Liberia, Palestine, Sudan, Ukraine, Afghanistan, Myanmar and other countries. ^1– 2, 4– 8^ However, the language used to describe these events often remains broad, and mostly context specific. Words such as disruption, adaptation and resilience are useful, but they make destruction sounds as temporary, accidental or educationally contained. It doesn’t capture the long-term public health damage and the implications on global medical capacity. This opinion article argues that medical education requires a sharper conceptual language that captures both.

## Why existing language is not enough?

Scholarship and advocacy on attacks on education have developed powerful terms such as educide and scholasticide to describe the large-scale destruction of educational systems. ^9– 11^ These concepts are valuable because they shift attention from isolated damage to patterned attacks on knowledge, continuity and collective futures. Despite their huge importance, these concepts don’t capture medical education distinctive role and how its destruction means populations elimination, due to the massive damage to healthcare capacities, current and future medical assets.

When a university is destroyed, a society loses learning, research capacity, livelihoods and intellectual life. When a medical school is destroyed, the society loses those but also loses a mechanism for producing clinicians, maintaining standards of safe practice, sustaining specialist services and rebuilding health systems during and after war. Teaching hospitals are simultaneously care institutions and educational institutions; faculty members are simultaneously academics, clinicians and supervisors; and medical students are learners who will become future doctors. ^12, 13^ The destruction of medical education therefore blurs the boundary between attacks on education and attacks on health. Furthermore, educators and students in conflict settings often show extraordinary commitment by moving teaching online, relocating clinical placements, relying on diaspora faculty, or improvising assessments. These efforts deserve recognition. Yet resilience can become an ethically problematic frame if it celebrates survival while underplaying the responsibility of those who produce the conditions of destruction. In prolonged war, resilience may be less a sign of sustainable innovation than evidence of chronic improvisation under coercion.

## Defining meducide

I propose Meducide (M [edical]-edu [cation]-cide) as a framework that captures the systematic destruction, dismantling or incapacitation of medical education systems during armed conflicts, producing durable harm to health workforce formation, health system capacity and long-term population health. Meducide includes:

• destruction, occupation, militarisation or looting of medical schools, laboratories, libraries and teaching hospitals;
• interruption of supervised clinical placements, procedural training and longitudinal patient contact;
• collapse or fragmentation of accreditation, licensing, assessment, admissions and professional regulation;
• detention, killing, displacement, silencing or forced migration of students, educators, clinicians and academic leaders;
• political interference in curricula, faculty appointments or access to medical education, including gendered or discriminatory exclusion;
• structural conditions, such as siege, blockade, sanctions, insecurity, electricity failure or digital isolation, that make medical education impossible to sustain.

This definition deliberately includes direct and indirect mechanisms. Some medical schools may be deliberately targeted because of their symbolic, political or institutional importance. Others may be rendered non-functional through the cumulative effects of insecurity, destroyed infrastructure, governance collapse, and loss of staff. In both situations, the educational and public health outcome can be similar; the erosion of current and future clinical capacity.

## What does meducide visualise?

• Clinical training bottleneck:

Studies from conflict-affected settings show that students may continue studying while losing access to the very clinical experiences that make them competent and safe practitioners. ^1, 3, 14^ Therefore, a medical education system can appear to be operating, while the core of professional formation is being hollowed out.

• Infrastructure of standards:

Medical education depends on governance systems that are less visible than buildings but equally essential. These include: accreditation, licensing examinations, records, curricular oversight, postgraduate pathways and professional regulation. Armed conflicts fragment and suspend these systems. Students may be displaced without recognised credits; examinations may be delayed; curricula may diverge across competing authorities; and graduates may struggle to prove competence or transfer qualifications. The result risks patient safety, accountability, quality, and workforce planning.

• Intergenerational workforce harm

War kills, displaces or/and exhausts practicing clinicians. Faculty loss is especially consequential because academic clinicians train both current students, junior doctors and future educators. When they leave, killed, detained, or forced into full-time emergency service, a country loses teachers, future supervisors, examiners, researchers and experts. ^12, 13, 15^ When added to health workforce shortages, this creates a compounding cycle; weakened training leads to fewer graduates, which leads to weaker services, which leads to further reduce training capacity.

• Political determinants of health

The destruction of medical education is not only an educational outcome of war; it is also a political determinant of health. It shapes who can become a doctor, which populations will have access to care, which will have access to life, which specialties survive, and whether a health system can recover without long-term dependency on external actors. This is why medical schools should be understood as infrastructures of care and sovereignty, not simply as universities that happen to teach medicine.

## Careful use of Meducide 

A new concept or framework can clarify, but it can also overreach. Meducide should not be used as a rhetorical substitute for documentation. Not every damaged medical school was necessarily targeted because it was a medical school; not every interruption demonstrates intent; and not every adaptive response represents recovery. The framework is strongest when used with evidence about mechanisms, actors, duration and consequences. It should invite more precise documentation, not less. Therefore, a balanced use of Meducide should distinguish between direct attacks, indirect incapacitation and structural erosion. It should also avoid treating all conflict-affected settings as identical. The educational consequences of bombardment, occupation, siege, sanctions, gender exclusion, civil war, forced displacement and digital collapse overlap, but they are not the same. The value of Meducide is not that it flattens these differences. Its value is that it holds together a common public health consequence: the destruction of the capacity to educate future clinicians.

## What should change?

Naming Meducide should change medical education policy and humanitarian practice.

First, monitoring systems should document attacks on medical education separately from general attacks on higher education. Documentation should include physical destruction, loss of teaching hospitals, faculty displacement, interruption of licensing, loss of records, student displacement and the collapse of clinical placements.

Second, humanitarian and reconstruction planning should treat medical education as critical health infrastructure. Rebuilding hospitals without rebuilding the institutions that train staff risks producing dependency rather than recovery.

Third, international medical education organisations, regulators and universities should develop conflict-contingent mechanisms for continuity and recognition. These may include emergency credit transfer, temporary visiting student status, supervised cross-border placements, remote teaching by diaspora faculty, low-bandwidth learning resources, and assessment arrangements that protect standards without punishing students for circumstances beyond their control.

Fourth, medical curricula should include conflict medicine, disaster response, trauma care, triage, ethics under scarcity, psychological first aid and human rights. This prepares students to support communities and colleagues in conflict-affected areas, and prepares them to manage future crises in their countries. However, this must be done carefully, because the purpose is not to normalise war as an expected learning environment, but to prepare future clinicians to practise ethically when institutions are under threat and populations are exposed to extreme harm. ^14, 16^

## The professional responsibility for global medical education

Medical education communities often describe themselves as global, collaborative and committed to social accountability. Meducide evaluates the seriousness of such commitments. Solidarity shouldn’t limited to statements of concern after destruction has occurred. It should include durable partnerships, reciprocal faculty networks, protected pathways for displaced students, support for local leadership, and advocacy for the protection of teaching hospitals and medical schools.

This responsibility also requires humility. International support can reproduce dependency if it substitutes for local governance or treats conflict-affected institutions only as recipients of aid. Therefore, effective partnerships should strengthen local accreditation, faculty pipelines, research capacity and decision-making, rather than creating parallel systems that disappear when donor priorities change. ^4, 17, 18^

## Conclusion

Destruction of medical education during wars is more than educational disruption; it damages health systems and population health for generations. Medical schools, teaching hospitals, and accreditation systems are infrastructures of future care. When destroyed, occupied, fragmented, or inaccessible, their loss affects patients long after war ends. Meducide names this harm, helping researchers document it, policymakers recognise medical education as health infrastructure, and educators act before improvisation becomes the only response. Protecting medical education is a public health, human rights, and peacebuilding imperative.

## Data Availability

No data are associated with this paper.
