# Supplementary material for: Meducide - Towards a new conceptual framework for the destruction of medical education in armed conflicts
Source: MedEdPublish (2016). 2026 Jun 18;16:23. [Version 1] doi: 10.12688/mep.21604.1 (PMC13560647; doi:10.12688/mep.21604.1)
Supplement: Peer review history

# Reviewer report: Reviewer response for version 1

**DOI:** 10.21956/mep.23149.r46305

**Published:** 2026-08-04

**Authors:** Rahul Barmanray

**Affiliations:** 1 The Royal Melbourne Hospital, Melbourne, Australia

This article summarises the particular effects of conflict on medical education and how these differe from those on education more broadly, including in scope, duration, and consequences. This is a potentially valuable contribution to the literature as a conversation starter.

 The paper states a number of considerations in application of the term but does so sufficiently vaguely that the term is likely to be used in the flattening way the author was keen to avoid. The risk of this could be reduced by greater specificity being included in the section "Careful use of Meducide".

 The term "Meducide", while defined carefully in the paper, is likely to be understood as the killing of medical personnel. I propose the slightly unwieldy but unambiguous "Mededucide" or "Med-educide", with a greater sematic link to the previously defined term of "educide" and an unambiguous use of the "Med-" qualifier.

Is the topic of the opinion article discussed accurately in the context of the current literature?

Yes

Are arguments sufficiently supported by evidence from the published literature?

Yes

Are all factual statements correct and adequately supported by citations?

Yes

Are the conclusions drawn balanced and justified on the basis of the presented arguments?

Yes

Reviewer Expertise:

Medical education, Diabetes, Endocrinology, Digital Health

I confirm that I have read this submission and believe that I have an appropriate level of expertise to confirm that it is of an acceptable scientific standard.

# Reviewer report: Reviewer response for version 1

**DOI:** 10.21956/mep.23149.r46089

**Published:** 2026-06-29

**Authors:** Ayad M Al-Moslih

**Affiliations:** 1 University of Central Lancashire, Preston, England, UK

Dear Author, and respected editorial team, Thanks for sharing this opinion article which proposes the neologism "meducide" to describe the systematic destruction of medical education during armed conflicts. The author successfully argues that this destruction represents a distinct, long-term public health crisis rather than a secondary educational disruption. The manuscript is conceptually robust, well-reasoned, and adds highly valuable vocabulary to the intersection of medical education, humanitarian health, and conflict studies. My review offers no fundamental criticism of this current account; rather, it aims to view the framework through different lenses to enrich the manuscript's actionable outcomes.

 I have no major concerns regarding the fundamental validity of the arguments or conclusions presented in this manuscript. I am rather keen to pose some thoughts to foster critical thinking and invite broader perspectives.

 The topic is discussed accurately within the context of the current literature; the author effectively grounds the new concept in existing scholarship on "educide" and "scholasticide" while clearly delineating the unique public health implications of destroying medical training pipelines. I particularly support the proposition of incorporating core competencies on disaster planning, preparedness, and response in health professions curricula in conflicts zones (Markenson et al, 2005)  

 Areas for Critical Reflection

 1. Under the section "Why existing language is not enough?", the author effectively contrasts meducide with the existing concepts of educide and scholasticide. However, it would strengthen the argument to briefly clarify how "meducide" intersects with existing frameworks regarding attacks on general healthcare facilities, especially since the author accurately notes that teaching hospitals function simultaneously as care and educational institutions.

 2. In the "Intergenerational workforce harm" section, the author notes that faculty loss creates a compounding cycle where weakened training leads to fewer graduates and weaker health services. While the text cites sources 12, 13, and 15 regarding workforce stock and post-conflict retention to support this, briefly elaborating on how these specific references document this compounding cycle would enhance the manuscript's evidentiary support.

 3. In the section "What should change?", the first recommendation suggests that monitoring systems should document attacks on medical education separately from general attacks on higher education. The manuscript would benefit from a sentence suggesting specific international monitoring bodies (e.g., WHO Surveillance System for Attacks on Health Care or the Global Coalition to Protect Education from Attack) that are best positioned to implement this distinct categorisation (Kapit-Spitalny & Burde; Novelli & Selenica, 2014; World Health Organization, 2019).

 4. In the "Intergenerational workforce harm" section, adding a brief discussion on the compounding disconnection to career growth norms would further illustrate the severe, long-term professional toll on future clinicians.

Is the topic of the opinion article discussed accurately in the context of the current literature?

Yes

Are arguments sufficiently supported by evidence from the published literature?

Yes

Are all factual statements correct and adequately supported by citations?

Yes

Are the conclusions drawn balanced and justified on the basis of the presented arguments?

Yes

Reviewer Expertise:

Preparedness in health professions education, Clinical Skills education, Disaster medical management

I confirm that I have read this submission and believe that I have an appropriate level of expertise to confirm that it is of an acceptable scientific standard.
